# oposSOM-Browser: an interactive tool to explore omics data landscapes in health science

**DOI:** 10.1186/s12859-020-03806-w

**Published:** 2020-10-19

**Authors:** Henry Loeffler-Wirth, Jasmin Reikowski, Siras Hakobyan, Jonas Wagner, Hans Binder

**Affiliations:** 1 Interdisciplinary Centre for Bioinformatics, Leipzig University, Härtelstraße 16-18, 04107 Leipzig, Germany; 2 Group of Bioinformatics, Institute of Molecular Biology, National Academy of Sciences, 7 Hasratyan str, 0014 Yerevan, Armenia; 3 LIFE, Leipzig Research Center for Civilization Diseases, Leipzig University, Philipp-Rosenthal-Straße 27, 04103 Leipzig, Germany

**Keywords:** Interactive data analysis, Transcriptomics, Results browser

## Abstract

**Background:**

oposSOM is a comprehensive, machine learning based open-source data analysis software combining functionalities such as diversity analyses, biomarker selection, function mining, and visualization.

**Results:**

These functionalities are now available as interactive web-browser application for a broader user audience interested in extracting detailed information from high-throughput omics data sets pre-processed by oposSOM. It enables interactive browsing of single-gene and gene set profiles, of molecular ‘portrait landscapes’, of associated phenotype diversity, and signalling pathway activation patterns.

**Conclusion:**

The oposSOM-Browser makes available interactive data browsing for five transcriptome data sets of cancer (melanomas, B-cell lymphomas, gliomas) and of peripheral blood (sepsis and healthy individuals) at www.izbi.uni-leipzig.de/opossom-browser.

## Background

Many bioinformatics tools are currently in transition from software libraries to interactive solutions designed for a broader user community including data scientists, output-oriented medical researchers and experimenters with needs in intuitive visualization and exploration for complex, multidimensional data. We here present an interactive web-tool which extends functionalities of our Bioconductor R-package ‘oposSOM’ designed to analyse transcriptome data in cancer and health research [1]. The method is based on self-organizing map (SOM) machine learning for dimension reduction, visualization, and comprehensive downstream analysis. This so-called ‘high-dimensional data portraying’ visualizes individual data landscapes, and performs function mining, modular feature selection, sample stratification, diversity analysis, and phenotype mapping [2]. It was applied to a series of data types (transcriptome, methylome, proteome, genome), diseases (cancers such as melanoma, lymphoma; autoimmune diseases) on the level of patient-cohort and cell system specimen (see, e.g., [3–5]). The method was so far used in more than 60 publications in a large variety of studies related to cellular development, toxicology, health studies, cell, and molecular biology. A full list of references can be found in Additional file 1: Appendix.

We further developed the analytic options of this method and here present an interactive browsing tool, which provides intuitive access to all the information generated by means of the data portraying method. The oposSOM-Browser complements and extends the functionalities of the oposSOM software package by interactive functionalities in the context of gene-expression and gene-function profiling, associations with phenotypes, and pathway activities in selected transcriptome data sets on different cancer entities and blood transcriptomes. The oposSOM-browser is hosted by the ‘Leipzig Health Atlas’, a sharing platform for publications, biomedical data, models, and software tools from the field of health research.

## Implementation

### Implementation and availability

The oposSOM-Browser is implemented using R-Shiny [6]. The Shiny web server is permanently running as Docker container hosted by the Leipzig Health Atlas app infrastructure. It can be accessed via any standard web browser under www.izbi.uni-leipzig.de/opossom-browser.

### Datasets

Five datasets are currently available in the oposSOM-Browser: (1) 917 specimen of germinal B cell lymphomas and selected healthy control samples [3], see below; (2) 80 melanoma and nevi samples [5]; (3) 137 low-grade gliomas [4]; (4) 180 blood samples of community acquired pneumonia patients [7]; and (5) 3388 blood samples collected from healthy participants of a population-based health study [8]. All data sets were pre-processed using oposSOM. Further data sets are presently in preparation for release in the browser. Interested users are invited to provide their analyses to the browser via request to the corresponding author.

### Modules

**Fig. 1:**
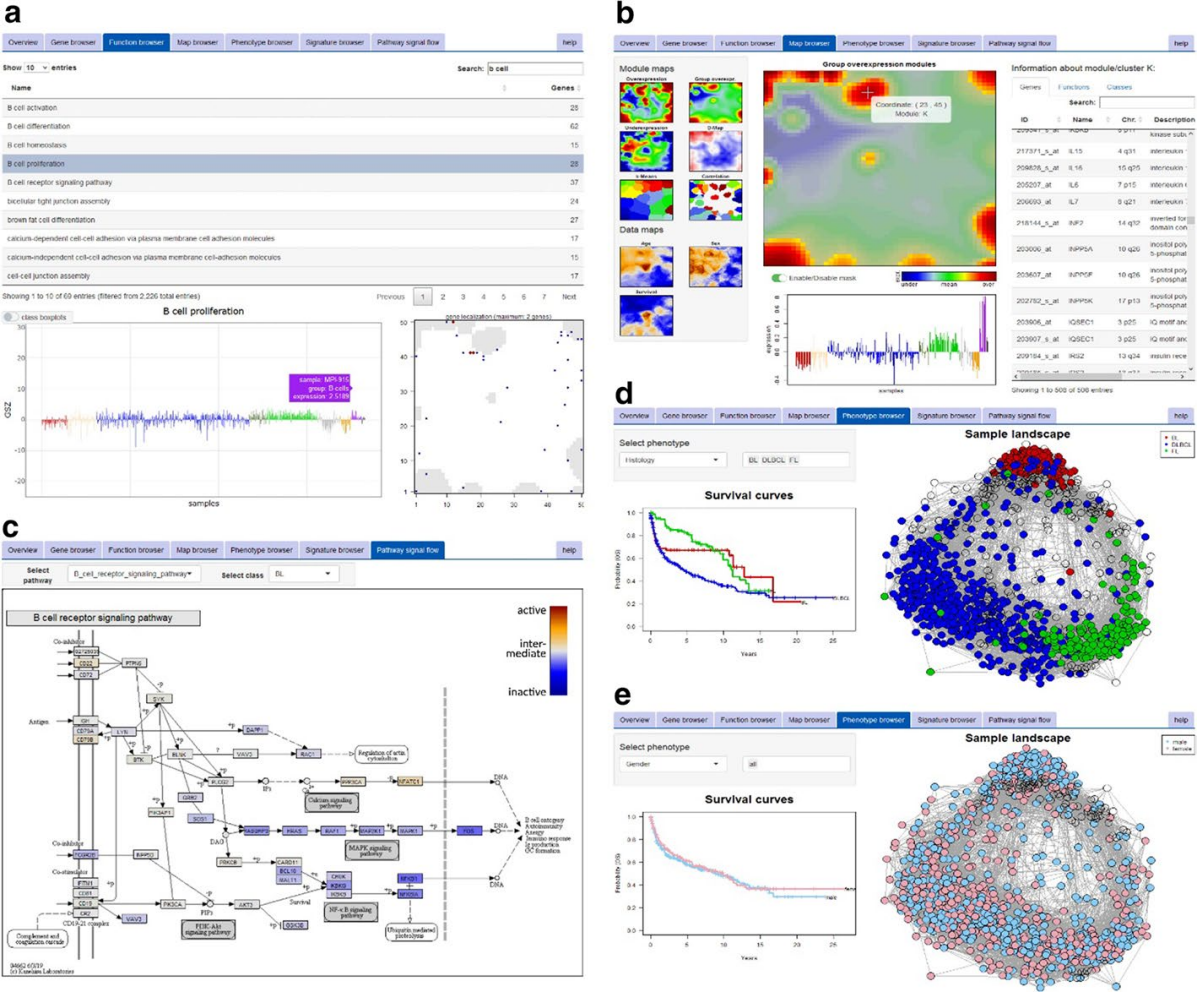
Screenshots illustrating different functionalities of the oposSOM browser using the B-cell lymphoma data set as example: **a** The ‘Function browser’ provides expression patterns of the GO set ‘B cell proliferation’. It reveals high activation of this cellular program in healthy B cells and follicular lymphomas (purple and green bars in profile plot, respectively), whereas Burkitt’s lymphomas show low activation (red bars). Genes of this set accumulate around module ‘K’ in the data landscape (bottom right map). **b** The expression profile of this module shows significant over-expression in healthy B cells. The table lists all 508 genes belonging to this module. **c** The KEGG pathway ‘B cell receptor signalling’ shows consistent inactivation in Burkitt’s lymphomas. **d** The mapping of samples according to their patho-histological phenotype reveals clear separation into three main subtypes with different prognoses. In contrast, stratification according to the patient’s gender results in virtually uniform scattering without differences of their overall survival curves (compare **d** and **e**)

Functionalities are arranged as browser-tabs as shown in Fig. 1. Detailed descriptions are provided in Additional file 1: Appendix and in the ‘Guided tour’ tab in the Browser:

- The ‘overview’ tab provides a description of the data set selected, a link to the corresponding publication, and additional information such as the dimensionality and version of oposSOM package used for data processing.
- The ‘gene browser’ and ‘function browser’ tabs provide tables of all up to 55,000 genes in the dataset and of all up to 10,000 functional gene sets considered. It enables the visualization of feature-profiles and mapping of the selected genes into the SOM data landscape.
- The ‘map browser’ tab provides an overview about patterns of the expression landscape: Lists of co-expressed genes are given together with enriched functional gene sets (for details see [2]). Accompanying data maps are shown for age, gender and prognosis (in terms of overall survival curves) of the individuals in the respective cohort.
- The ‘phenotype’ tab provides the correlation network of sample similarities. The network can be stratified considering up to 25 different phenotypes related to patient information, clinical or molecular characteristics, along with the corresponding survival curves.
- The ‘signature’ tab enables the user to upload lists of signature genes (Ensemble-IDs or gene names). The browser delivers their mean expression profile across all samples and shows their location in the SOM data landscape. Further, the provided signature is benchmarked (ROC and AUC) with regard to the phenotype classes selected.
- The ‘pathway signal flow’ tab shows KEGG signalling pathways with genes colour-coded according to their activity level [9].

## Results: use case lymphoma browser

Our use case presents a transcriptome dataset of 917 B cell lymphoma specimen and healthy control samples [3]. The oposSOM-browser provides a holistic view on the expression landscape, the heterogeneity of activated gene-regulatory programs and their association with different lymphoma subtypes and clinical phenotypes (see Fig. 1). A first step is to examine the expression landscape in a particular data set using the map browser (Fig. 1b), assigning the series of lymphoma subtypes and healthy cell references to the corresponding expression modules together with their functional interpretation. Then, the gene browser enables investigating genes of interest, e.g. by selecting frequently mutated genes or genes previously reported as expression markers of different lymphoma subtypes, by mapping them into the expression landscape and/or by exploring their expression profile across subtypes and samples. Patterns of cellular activity can be explored using the function and PSF browser tabs (Fig. 1a, c), in order to identify subtype-specific or ubiquitous processes and signalling cascades. Finally, mapping of clinical, genetic and phenotypic subtyping schemes enables the mutual comparison of different lymphoma strata in terms of cluster structure and of survival hazard ratios (see Fig. 1d, e for stratification based on patho-histological diagnosis and by gender, respectively).

## Conclusion

oposSOM-Browser is a novel tool for the interactive exploration of high-dimensional omics data and associated phenotypes, allowing interested researchers to browse through the data by addressing specific issues and their own questions in order to generate or to validate hypotheses not or incompletely considered before.

Further extension of available data sets will build a library of annotated omics landscapes for health science.

## Availability and requirements

- Project name: oposSOM-Browser
- Project home page: www.izbi.uni-leipzig.de/opossom-browser
- Operating systems: Platform independent
- Programming language: R using Shiny package
- Other requirements: Internet browser
- License: oposSOM-Browser is licensed under the terms of use defined for the Leipzig Health Atlas
- Restrictions to use by non-academics: license needed

## Supplementary information

**Additional file 1.** Appendix with reference list and guided tour.

## Data Availability

oposSOM-Browser is available under www.izbi.uni-leipzig.de/opossom-browser.

## Abbreviations

AUC: Area under the ROC curve
KEGG: Kyoto Encyclopedia of genes and genomes
PSF: Pathway signal flow
ROC: Receiver operating characteristic
SOM: Self-organizing map
