# Supplementary material for: oposSOM-Browser: an interactive tool to explore omics data landscapes in health science
Source: BMC Bioinformatics. 2020 Oct 19;21:465. doi: 10.1186/s12859-020-03806-w (PMC7574456; doi:10.1186/s12859-020-03806-w)
Supplement: Supplementary file 1 — Additional file 1. Appendix with reference list and guided tour. [file 12859_2020_3806_MOESM1_ESM.pdf]

oposSOM-Browser: An interactive tool to explore data landscapes in health science

Henry Loeffler-Wirth[1], Jasmin Reikowski[1], Siras Hakobyan[2], Jonas Wagner[3] and Hans Binder[1]

[1] Interdisciplinary Centre for Bioinformatics, Leipzig University, Härtelstraße 16-18, 04107 Leipzig, Germany

[2] Group of Bioinformatics, Institute of Molecular Biology, National Academy of Sciences, 7 Hasratyan str, 0014 Yerevan, Armenia

[3] LIFE, Leipzig Research Center for Civilization Diseases, Leipzig University, Philipp-Rosenthal-Straße 27, 04103 Leipzig, Germany

Supplementary text

1. List of publications using data portraying and oposSOM R-package 2
  1.1. Publications of Loeffler-Wirth, Binder et al. 2
  1.2. Publications of external research groups 4
2. Guided tour to oposSOM-Browser functionalities 5
  2.1. Overview 5
  2.2. Gene & function browser 6
  2.3. Map browser 8
  2.4. Phenotype browser 10
  2.5. Signature browser 11
  2.6. Pathway signal flow (PSF) browser 12

1. List of publications data portraying using oposSOM

1.1. Publications of Loeffler-Wirth, Binder et al.

1) Wirth et al.: Expression cartography of human tissues using self-organizing maps. *BMC Bioinformatics 2011*
2) Binder et al.: Molecular phenotypic portraits - exploring the ‘OMEs’ with individual resolution. *HIBIT conference 2011 proceedings*
3) Binder et al.: Genomic and molecular phenotypic portraits – exploring the ‘OMes’ with individual resolution. *IEEE Xplore, Health Informatics and Bioinformatics (HIBIT) 2011*
4) Binder et al.: Portraying high-dimensional OMICs data with individual resolution. *CAMDA conference 2011 proceedings*
5) Wirth et al.: MALDI-typing of infectious algae of the genus Prototheca using SOM portraits. *Journal of Microbiological Methods 2012*
6) Wirth et al.: Mining SOM expression portraits: Feature selection and integrating concepts of molecular function. *BioData Mining 2012*
7) Steiner et al.: A global genome segmentation method for exploration of epigenetic patterns. *PLOS ONE 2012*
8) Hopp et al.: Portraying the Expression Landscapes of B-Cell Lymphoma - Intuitive Detection of Outlier Samples and of Molecular Subtypes. *Biology 2013*
9) Hopp et al.: Portraying the expression landscapes of cancer subtypes: a glioblastoma multiforme and prostate cancer case study. *Systems Biomedicine 2013*
10) Binder et al.: Analysis of large-scale OMIC data using Self Organizing Maps. Encyclopedia of Information *Science and Technology 2014*
11) Binder et al.: Personalized disease phenotypes from massive OMICs data. *Big Data Analysis in Bioinformatics and Healthcare 2014*
12) Reifenberger et al.: Molecular characterisation of long-term survival with glioblastoma using genome- and transcriptome-wide profiling. *International Journal of Cancer 2014*
13) Charbord et al.: A Systems Biology Approach for Defining the Molecular Framework of the Hematopoietic Stem Cell Niche. *Cell stem cell 2014*
14) Binder et al.: Time-course human urine proteomics in space-flight simulation experiments – A high resolution and personalized machine learning analysis. *BMC Genomics 2014*
15) Wirth et al.: Analysis of miRNA expression using machine learning. *Methods in Molecular Biology 2014*
16) Cakir et al.: miRNA expression landscapes in stem cells, tissues and cancer. *Methods in Molecular Biology 2014*
17) Ahmad et al.: How Stemlike Are Sphere Cultures From Long-term Cancer Cell Lines? Lessons From Mouse Glioma Models. *Journal of neuropathology and experimental neurology 2014*
18) Weller et al: Molecular classification of diffuse cerebral WHO grade II/III gliomas using genome- and transcriptome-wide profiling improves stratification of prognostically distinct patient groups. *Acta Neuropathologica 2015*
19) Hopp et al.: Epigenetic heterogeneity of B-cell lymphoma: DNA methylation, gene expression and chromatin states. *Genes 2015*
20) Hopp et al.: Epigenetic heterogeneity of B-cell lymphoma: Chromatin modifiers. *Genes 2015*
21) Hopp et al.: Function shapes content: DNA-methylation marker genes and their impact for molecular mechanisms of glioma. *Journal of Cancer Research Updates 2015*

22) Nersisyan et al.: Gene set- and pathway- centered knowledge discovery assigns transcriptional activation patterns in brain, blood and colon cancer - A bioinformatics perspective. *International Journal of Knowledge Discovery in Bioinformatics 2016*
23) Rohanisarvestani et al.: Generation of human induced pluripotent stem cells using non-synthetic mRNA. *Stem Cell Research 2016*
24) Arakelyan et al.: Cartography of pathway signal perturbations identifies distinct molecular pathomechanisms in malignant and chronic lung diseases. *Frontiers in Genetics 2016*
25) Hamidouche et al.: Bistable epigenetic states explain age-dependent decline in mesenchymal stem cell heterogeneity. *Stem Cells 2016*
26) Gerber et al.: Mapping heterogeneity in a patient-derived melanoma culture by single-cell RNA-seq. *Oncotarget 2017*
27) Camp et al.: Multilineage communication regulates human liver bud development from pluripotency. *Nature 2017*
28) Cakir et al.: Dysregulated signal propagation in a MYC-associated Boolean gene network in B-cell lymphoma. *Biology, Engineering and Medicine 2017*
29) Binder et al.: Genomic and transcriptomic heterogeneity of colorectal tumours arising in Lynch syndrome. *Journal of Pathology 2017*
30) Arakelyan et al.: Autoimmunity, autoinflammation and inflammation: a systems view on signaling pathway deregulation profiles. *PLOS ONE 2017*
31) Pastushkova et al.: Analysis of the effects of different salt consumption levels on the urine protein composition during a 105-day isolation using the opoSOM program. *Human Physioloy 2017*
32) Cakir et al.: Dysregulated Signal Propagation in a MYC-associated Gene Network in B-cell Lymphoma. *Biology, Engineering and Medicine 2017*
33) Loeffler-Wirth et al.: Pseudotime dynamics in melanoma single-cell transcriptomes reveals different mechanisms of tumor progression. *Biology 2018*
34) Hopp et al.: Combined SOM-portrayal of gene expression and DNA methylation disentangles modes of epigenetic regulation in glioma. *Epigenomics 2018*
35) Kunz et al.: RNA-seq analysis identifies different transcriptomic types and developmental trajectories of primary melanomas. *Oncogene 2018*
36) Hopp et al.: Footprints of Sepsis framed within Community Acquired Pneumonia in the blood transcriptome. *Frontiers in Immunology 2018*
37) Thalheim et al.: On the Cooperation between Epigenetics and Transcription Factor Networks in the Specification of Tissue Stem Cells. *Epigenomes 2018*
38) Nikoghosyan et al.: Population levels assessment of the distribution of disease associated variants with emphasis on Armenians - a machine learning approach. *Frontiers in Genetics 2019*
39) Binder et al.: DNA methylation, transcriptome and genetic copy number signatures of diffuse cerebral WHO grade II/III gliomas resolve cancer heterogeneity and development. *Acta Neuropathologica Communications 2019*
40) Loeffler-Wirth et al.: A modular transcriptome map of mature B-cell lymphomas. *Genome Medicine 2019*
41) Arakelyan et al.: Transcriptome-guided drug repositioning. *Pharmaceutics 2019*
42) Bilz et al.: Teratogenic Rubella Virus Alters the Endodermal Differentiation Capacity of Human Induced Pluripotent Stem Cells. *Cells 2019*
43) Nikoghosyan et al.: SOMmelier– Intuitive visualization of the topology of grapevine genome landscapes using artificial neural networks. *Genes 2020*
44) Schmidt et al.: Portrayal of the human blood transcriptome in a population cohort and its relation to ageing and health. *Frontiers in Big Data* 2020

1.2. Publications of external research groups

1) Cheaib et al.: Epigenetic regulation of serotype expression antagonizes transcriptome dynamics in Paramecium tetraurelia. *DNA Research 2015*
2) James et al.: Multiparameter Analysis of Human Bone Marrow Stromal Cells Identifies Distinct Immunomodulatory and Differentiation-Competent Subtypes. *Stem Cell Reports 2015*
3) De Simone,M. et al.: Transcriptional Landscape of Human Tissue Lymphocytes Unveils Uniqueness of Tumor-Infiltrating T Regulatory Cells. *Immunity 2016*
4) Schwarzer et al.: The non-coding RNA landscape of human hematopoiesis and leukemia. *Nature Communications 2017*
5) Hutchins et al.: Models of global gene expression define major domains of cell type and tissue identity. *Nucleic Acids Research 2017*
6) Lukassen et al. Single-cell RNA sequencing of adult mouse testes. *Scientific data 2018*
7) Xie et al.: Single-Cell Deconvolution of Fibroblast Heterogeneity in Mouse Pulmonary Fibrosis. *Cell reports 2018*
8) Florian et al.: Aging Alters the Epigenetic Asymmetry of HSC Division. *PLoS Biology 2018*
9) Rennert et al.: The Diurnal Timing of Starvation Differently Impacts Murine Hepatic Gene Expression and Lipid Metabolism – A Systems Biology Analysis Using Self-Organizing Maps. *Frontiers in Physiology 2018*
10) Sanchez et al.: Morphological and transcriptomic analyses reveal three discrete primary stages of postembryonic development in the common fire salamander, Salamandra salamandra. *Journal of Experimental Zoology 2018*
11) Li et al.: Genomic and transcriptional Profiling of tumor infiltrated CD8 + T cells revealed functional heterogeneity of antitumor immunity in hepatocellular carcinoma. *Oncoimmunology 2018*
12) Kesch et al.: Correlation between genomic index lesions and mpMRI and 68Ga-PSMA-PET/CT imaging features in primary prostate cancer. *Scientific Reports 2019*
13) Fischer et al.: Signals trigger state-specific transcriptional programs to support diversity and homeostasis in immune cells. *Science Signalling 2019*
14) Xue et al.: A 3D Atlas of Hematopoietic Stem and Progenitor Cell Expansion by Multi-dimensional RNA-Seq Analysis. *Cell Reports 2019*
15) Locati et al.: Mining of self-organizing map gene-expression portraits reveals prognostic stratification of HPV-positive head and neck squamous cell carcinoma. *Cancers 2019*
16) Ma et al.: Single-cell RNA Sequencing of Lung Adenocarcinoma Reveals Heterogeneity of Immune Response-Related Genes. *JCI Insight 2019*
17) Herberg et al.: Loss of Msh2 and a single-radiation hit induce common, genome-wide, and persistent epigenetic changes in the intestine. *Clinical Epigenetics 2019*
18) Sabino-Pinto et al.: The Role of Plasticity and Adaptation in the Incipient Speciation of a Fire Salamander Population. *Genes 2019*
19) Zamudio et al.: AP-1 imprints a reversible transcriptional programme of senescent cells. *Nature Cell Biology 2020*
20) Malovichko et al.: Transcriptomic Insights into Mechanisms of Early Seed Maturation in the Garden Pea (Pisum sativum L.). *Cells 2020*
21) Kreuz et al.: ProstaTrend—A Multivariable Prognostic RNA Expression Score for Aggressive Prostate Cancer. European Urology 2020

2. Guided tour to oposSOM-Browser functionalities

2.1. Overview

The different data sets provided by oposSOM-Browsers are accessed via a select box in the top panel, which also includes version and citation information, and the main navigation menu (Figure S 1). The overview tab gives general information about the data set currently selected, such as dimensionality and version of oposSOM package used for data processing.

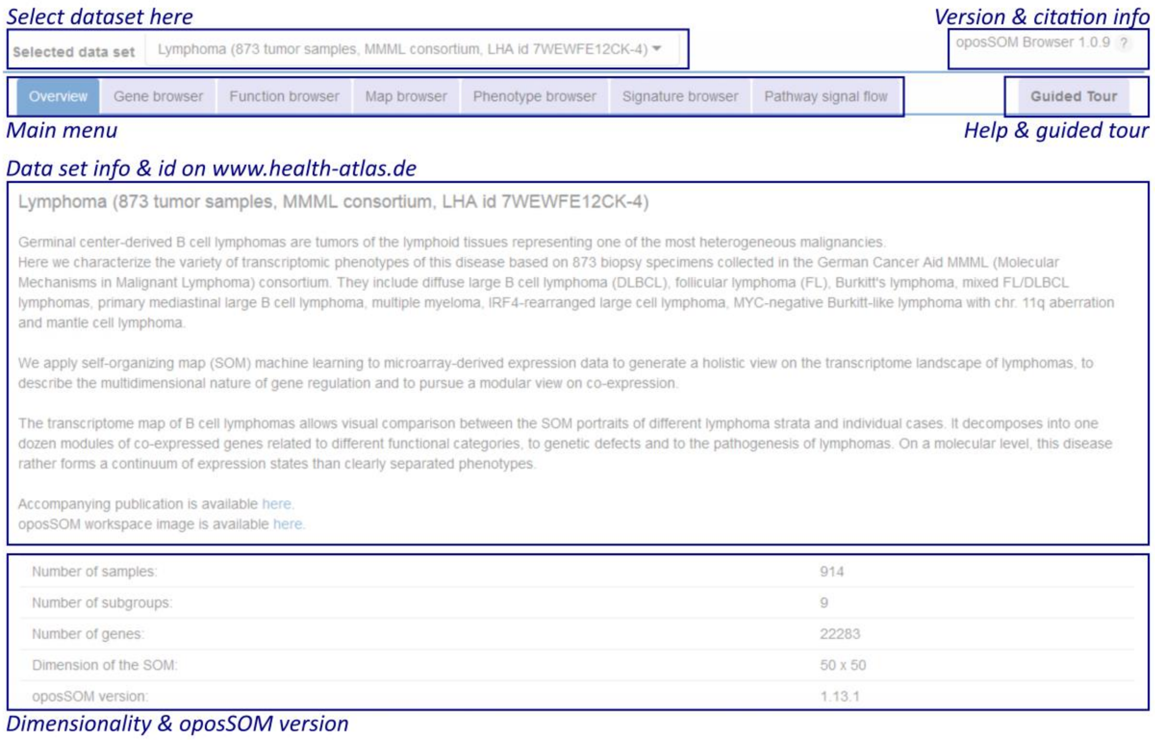

Figure S 1: Overview tab and main navigation of oposSOM-Browser

2.2. Gene & function browser

The gene (Figure S 2) and function (Figure S 3) browsers are designed in analogous layouts. They provide tables of all genes and functional gene sets available in the data set, respectively. These tables can be searched for gene and set names, Ensemble-IDs, and terms in the description. After selection of a table row, the expression profile and the mapping of the gene or gene set into the data landscape are generated. The profile plot can be toggled between a single sample barplot and a subgroup-wise boxplot.

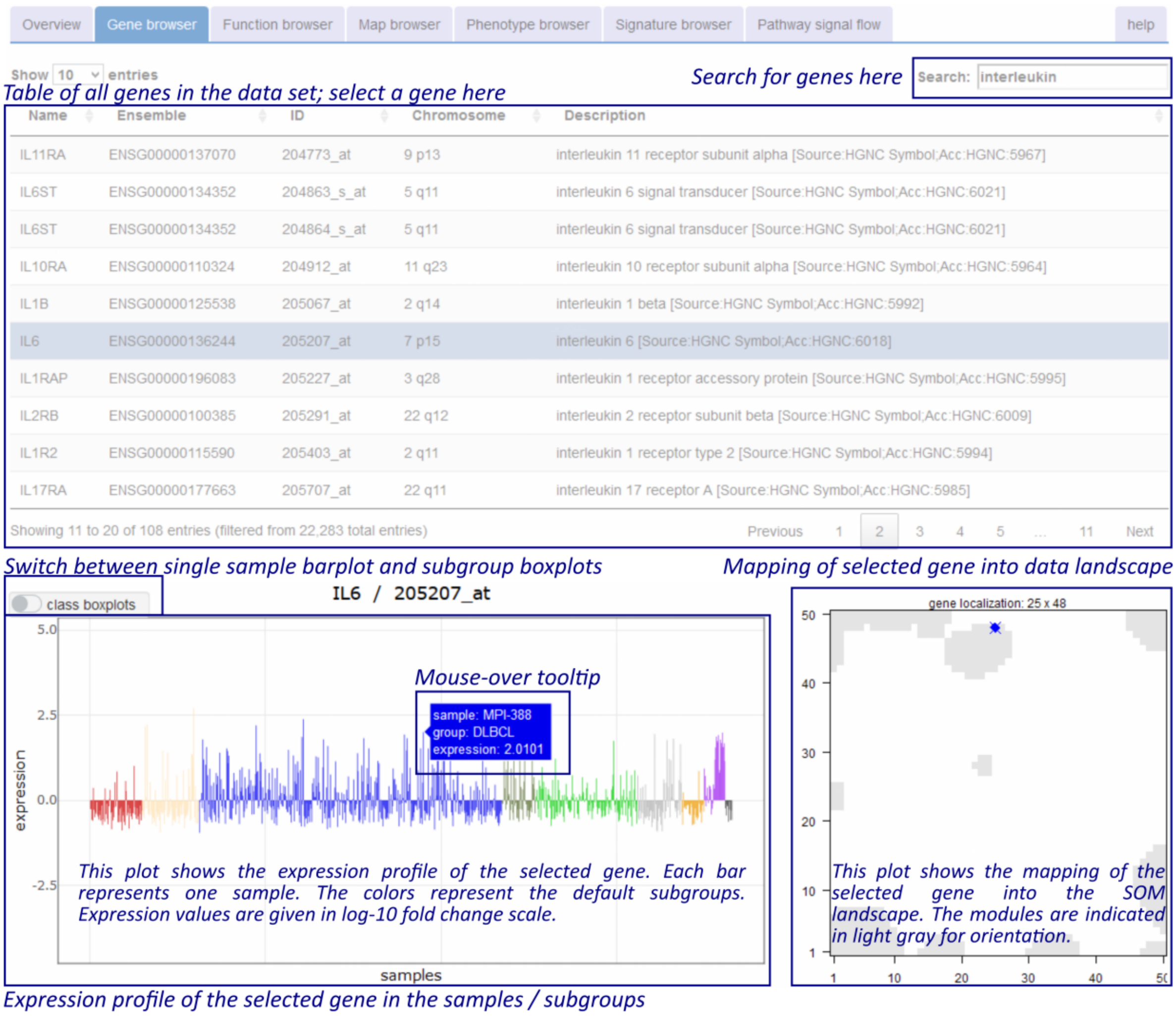

Name	Ensemble	ID	Chromosome	Description
IL11RA	ENSG00000137070	204773_at	9 p13	interleukin 11 receptor subunit alpha [Source:HGNC Symbol;Acc:HGNC:5967]
IL6ST	ENSG00000134352	204863_s_at	5 q11	interleukin 6 signal transducer [Source:HGNC Symbol;Acc:HGNC:6021]
IL6ST	ENSG00000134352	204864_s_at	5 q11	interleukin 6 signal transducer [Source:HGNC Symbol;Acc:HGNC:6021]
IL10RA	ENSG00000110324	204912_at	11 q23	interleukin 10 receptor subunit alpha [Source:HGNC Symbol;Acc:HGNC:5964]
IL1B	ENSG00000125538	205067_at	2 q14	interleukin 1 beta [Source:HGNC Symbol;Acc:HGNC:5992]
IL6	ENSG00000136244	205207_at	7 p15	interleukin 6 [Source:HGNC Symbol;Acc:HGNC:6018]
IL1RAP	ENSG00000196083	205227_at	3 q28	interleukin 1 receptor accessory protein [Source:HGNC Symbol;Acc:HGNC:5995]
IL2RB	ENSG00000100385	205291_at	22 q12	interleukin 2 receptor subunit beta [Source:HGNC Symbol;Acc:HGNC:6009]
IL1R2	ENSG00000115590	205403_at	2 q11	interleukin 1 receptor type 2 [Source:HGNC Symbol;Acc:HGNC:5994]
IL17RA	ENSG00000177663	205707_at	22 q11	interleukin 17 receptor A [Source:HGNC Symbol;Acc:HGNC:5985]

Figure S 2: Gene browser

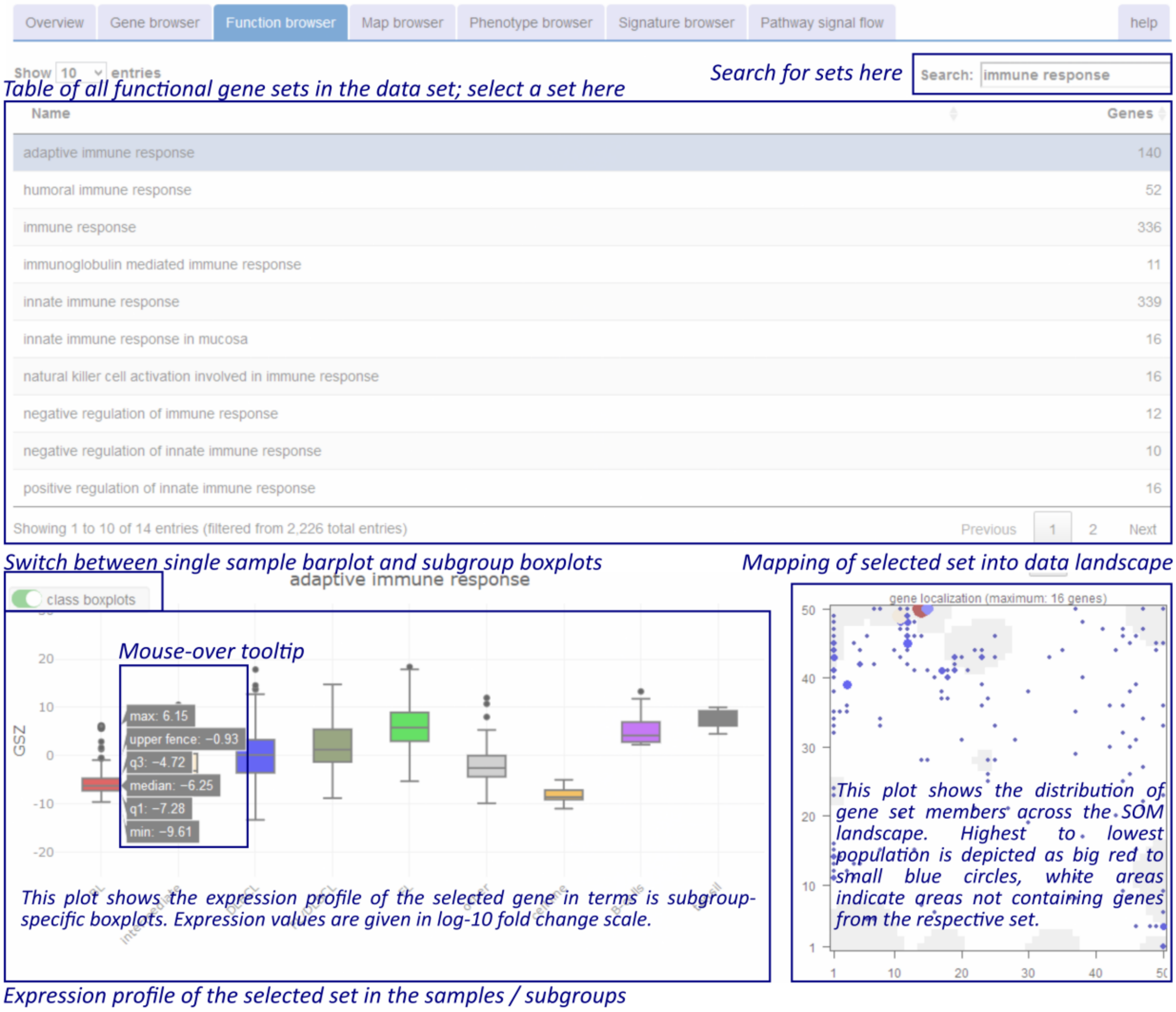

Figure S 3: Function browser

2.3. Map browser

The map browser provides information about expression modules detected in the data landscape, and of mapping of age, gender, and survival information into the data landscape. Module and data maps can be selected on the left-hand side (Figure S 4).

In the module maps, individual expression modules can be selected in the center frame, which will generate the corresponding expression profile across all samples of the study (Figure S 4). Additionally, the table on the right-hand side lists – depending on the selected tab - all genes in the module, enriched functional gene sets, or the subgroups which show high expression of this module, respectively.

In the data maps, single metagenes (i.e. pixels in the mosaic map) can be selected (Figure S 5). Additional information will be shown for all participants expressing this metagene: In the age map, age distribution of the corresponding participants will be shown in terms of a histogram, in the sex map a pie chart, and in the survival map curves of overall survival of patients expressing this metagene compared to those not expressing the metagene. The right-hand table lists all genes and enriched functional gene sets in the metagene, and the subgroups of the participants expressing this metagene, respectively.

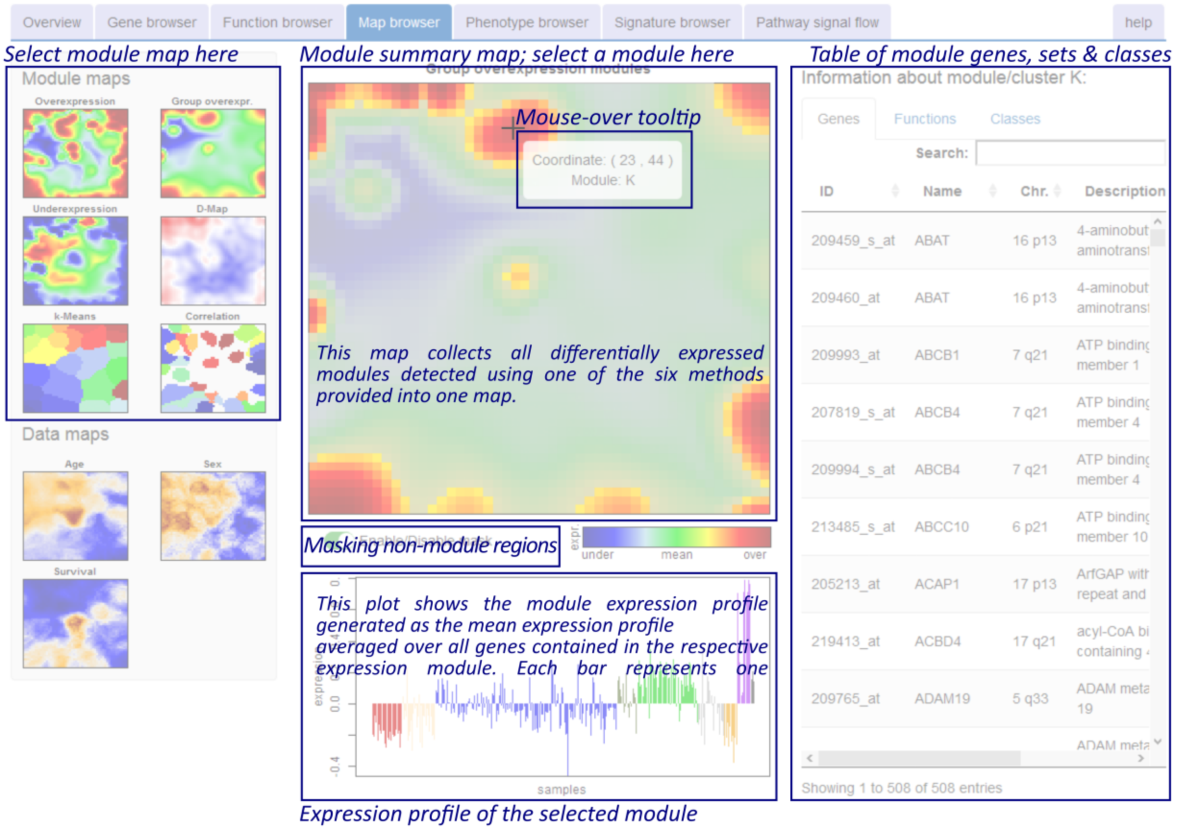

Figure S 4: Map browser, selection of a module map

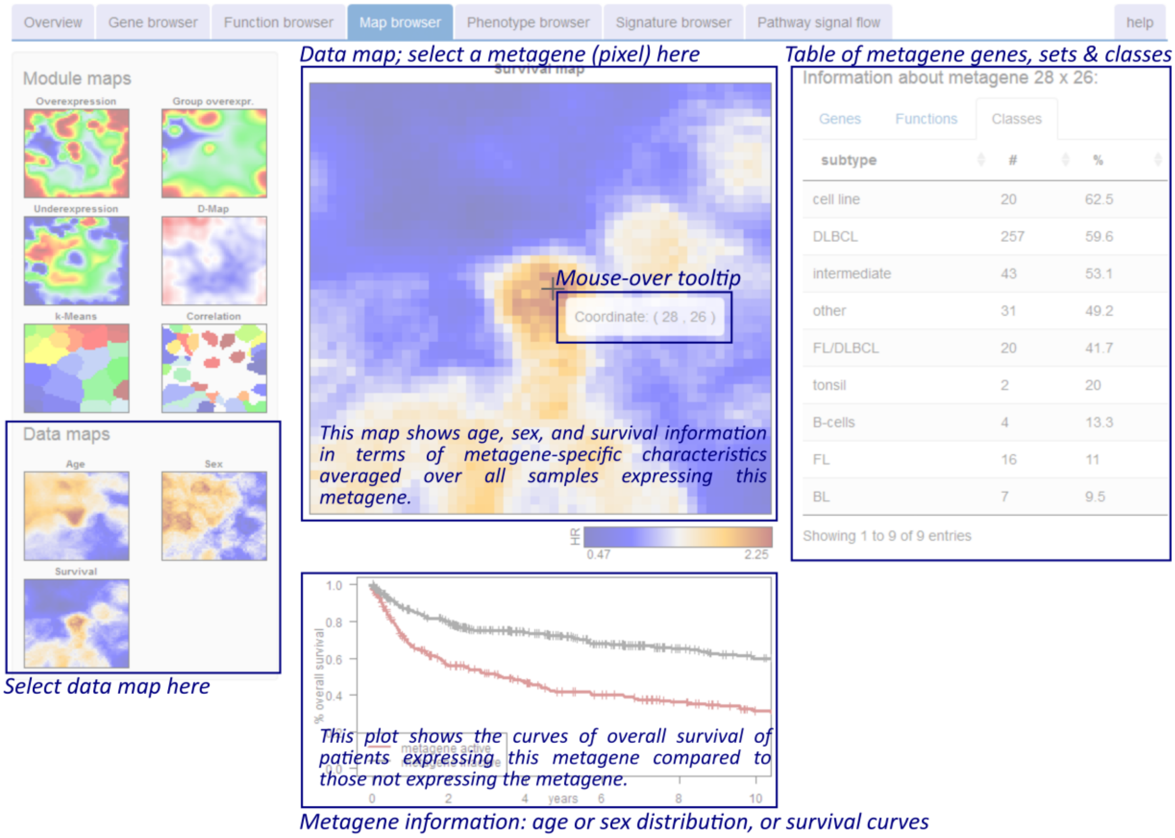

Figure S 5: Map browser, selection of a data map

2.4. Phenotype browser

The phenotype browser allows for the evaluation of different phenotypic stratifications with regard to the sample landscape and to prognostic impact (Figure S 6). General, clinical, or molecular phenotypes can be selected in the top left menu, where also individual phenotype characteristics can be added or removed.

The sample landscape, a correlation network of all samples in the data set, is then colored according to the selection. Clicking into one of the network nodes will open a frame showing the sample expression portrait along with patient age and gender if available.

Curves of overall survival as function of time are also generated given this information is available in the data set. They are stratified by the selected phenotype characteristics to evaluate associated diversification of prognosis.

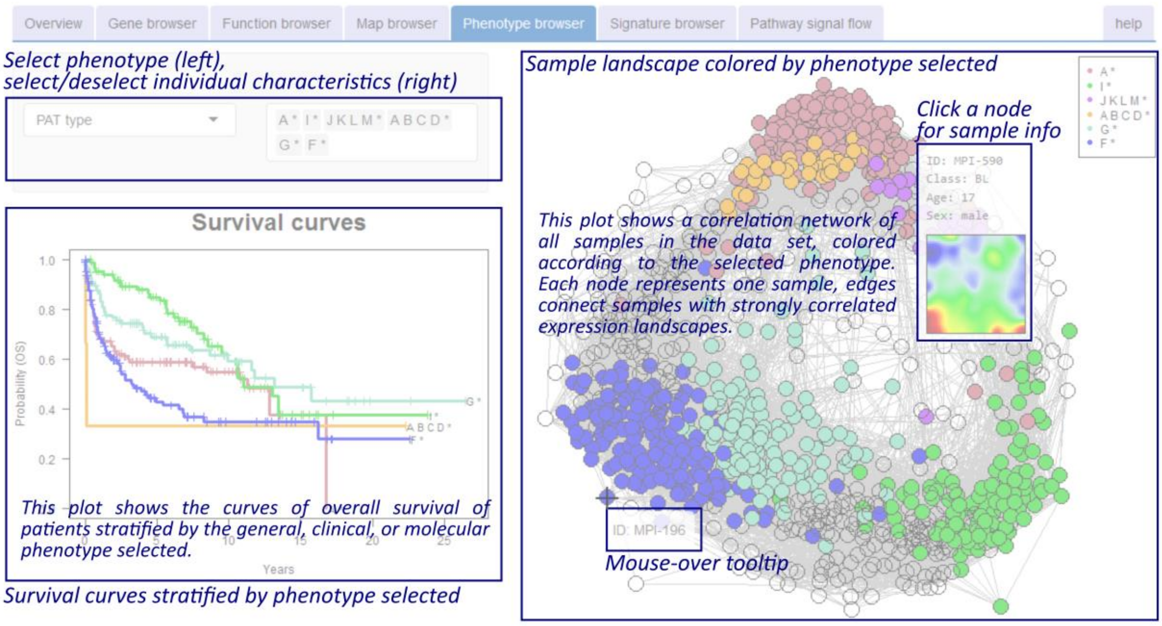

Figure S 6: Phenotype browser

2.5. Signature browser

In the signature browser, individual lists of genes can be uploaded to benchmark classification using these genes (Figure S 7). Click the gear button to open the menu for uploading the signature genes. They can be provided as Ensemble-IDs or gene names. For a submitted gene signature, the expression profile across all samples and the mapping into the data landscape will be displayed.

The signature is then used to identify samples of the target class in a set of samples that comprises the target class and additional classes/characteristics as selected in the bottom left select boxes. After selection, a click on the 'Generate ROC' button will start benchmarking resulting in a ROC curve plot together with the corresponding AUC measure.

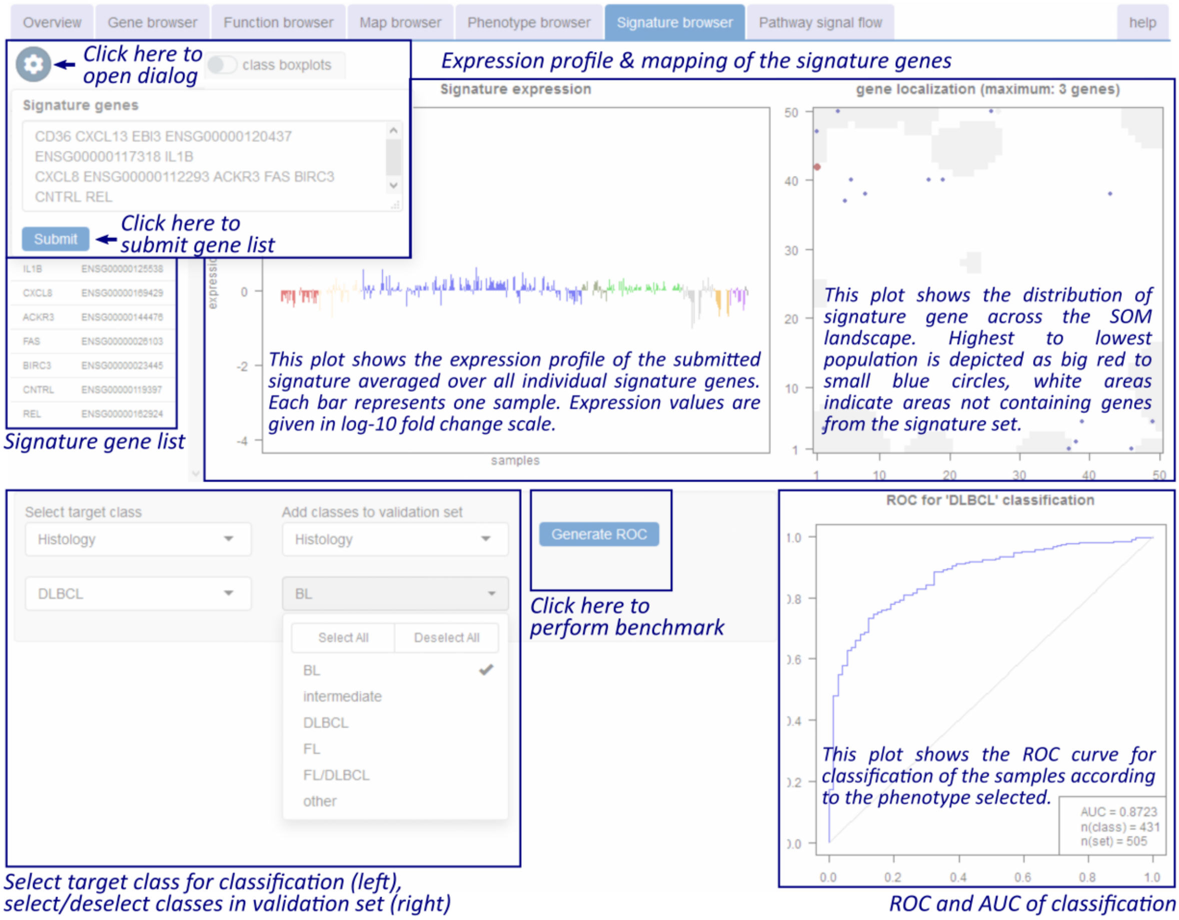

Figure S 7: Signature browser

2.6. Pathway signal flow (PSF) browser

Activation patterns in the KEGG cellular signalling pathways can be visualized using the pathway signal flow (PFS) browser (Figure S 8). The select boxes on top of the browser are used to access the pathways, and to select the subgroup for visualization of the signal flow in terms of color-coding the pathway nodes.

Hovering a node in the pathway will display the gene's position in the data landscape. Further, clicking a node shows a plot of signal flow values in the subtypes considered.

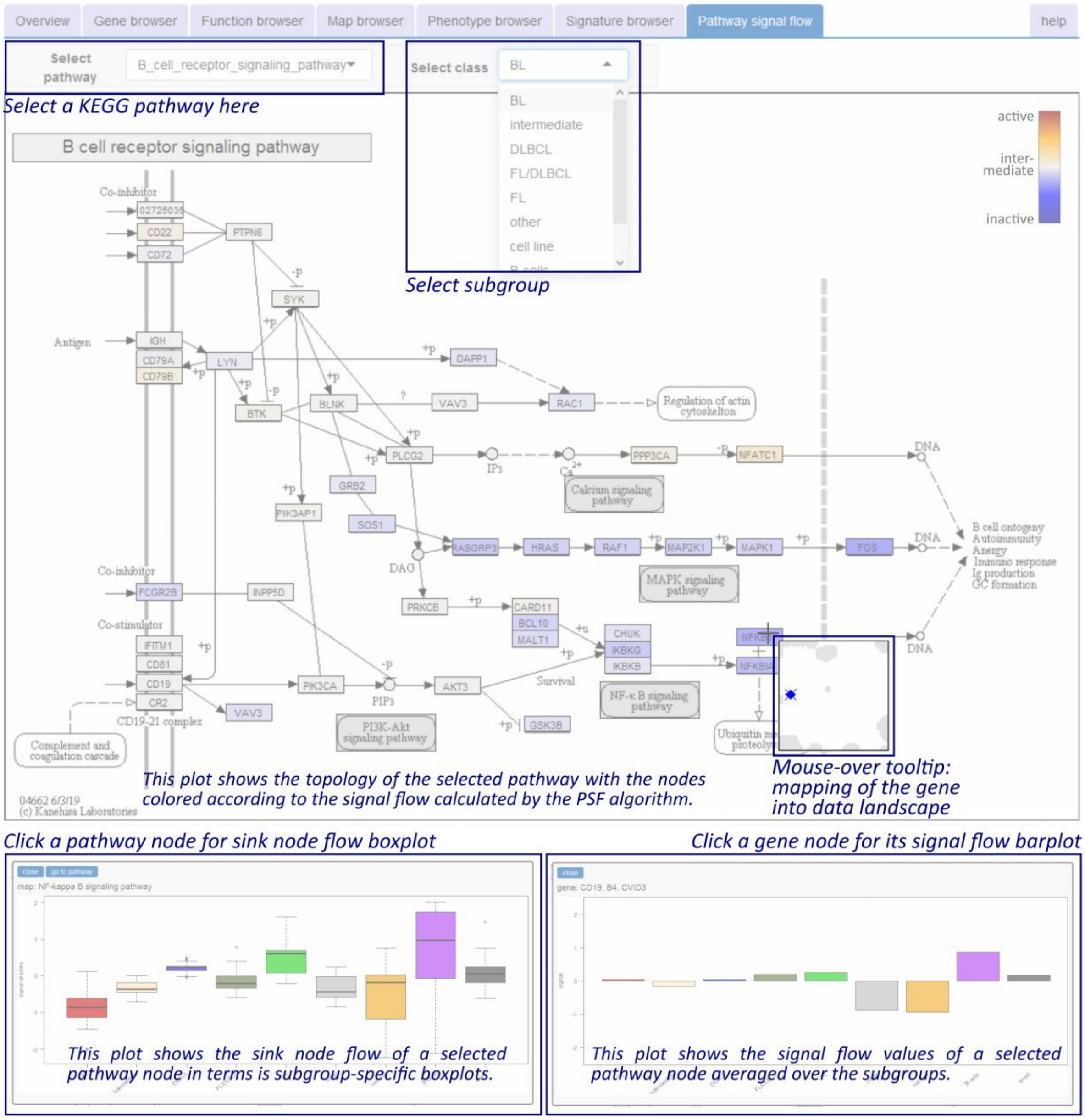

Figure S 8: PSF browser
